# Protective Effects of Bariatric Surgery on Kidney Functions by Inhibiting Oxidative Stress Responses Through Activating PPARα in Rats With Diabetes

**DOI:** 10.3389/fphys.2021.662666

**Published:** 2021-06-28

**Authors:** Hong-Wei Jiang, Yong Zhou, Pin-Yi Zhou, Tian-Yi Zhang, Jing-Yao Hu, Xue-Tao Bai

**Affiliations:** ^1^Department of General Surgery, The Fourth Affiliated Hospital of China Medical University, Shenyang, China; ^2^Department of Anaesthesiology, The Fourth Affiliated Hospital of China Medical University, Shenyang, China

**Keywords:** diabetic nephropathy, bariatric surgery, oxidative stress, inflammatory response, PPARα

## Abstract

**Objective:**

The aim of this study was to explore the protective effects and the regulatory mechanisms of bariatric surgery on kidney injury in diabetic rats.

**Methods:**

We established a useful type 2 diabetic rat model using high-fat and high-sugar diet feeding following low-dose streptozotocin (STZ) treatment. Sprague–Dawley (SD) rats were randomly divided into the following groups: control (Con) group, diabetic nephropathy (DN) group, and duodenal–jejunal bypass (DJB) surgery group. The food intake and body weight of rats were monitored and the glucose tolerance test (OGTT) test was performed every 2 weeks. The glomerular filtration rate (GFR) and urinary albumin excretion rate (UAFR) were measured to assess renal function. Hematoxylin–eosin (H&E), periodic acid–Schiff (PAS), and Masson staining were used to evaluate renal histopathological changes. TUNEL assay was performed to detect cell apoptosis. The expressions of oxidative stress factors and inflammatory factors in the renal tissues of rats were detected by ELISA. The expressions of PPARα, reactive oxygen species (ROS), and NF-κB were detected by immunofluorescence. For *in vitro* experiment, HK2 cells cultured with high glucose were treated with PPARα agonist, PPARα antagonist, and adenosine 5′-monophosphate (AMP)-activated protein kinase (AMPK) agonist. The expressions of AMPK/PPARα/NF-κB signaling pathway-related proteins were detected by Western blot.

**Results:**

Bariatric surgery improved the glucose tolerance of DN rats. The GFR was decreased, the promotion of urinary albumin excretion rate (UAER) was inhibited, and the renal injury was alleviated. The extracellular matrix fraction was decreased and the renal function was improved. Meanwhile, bariatric surgery activates PPARα, inhibits ROS release, reduces oxidative stress injury, and reduces renal cell apoptosis. *In vitro* experiment results showed that the AMPK activator could activate PPARα, downregulate NF-κB, and inhibit inflammatory response. The phosphorylation of AMPK was inhibited by PPARα antagonism.

**Conclusion:**

Bariatric surgery can activate PPARα, inhibit oxidative stress injury, and improve glucose metabolism and renal function in DN rats.

## Introduction

Diabetes mellitus (DM) has become a significant chronic disease that endangers people’s lives and health. Its clinical types include type I diabetes (T1DM), type II diabetes (T2DM), and gestational diabetes mellitus (GDM) (Guay and Regazzi, 2013). It is generally believed that the occurrence and development of T2DM are related to the patient’s lifestyle and genetic factors, of which obesity is an important independent risk factor, and more than 80% of T2DM patients are obese (Wilding, 2014). Diabetic nephropathy (DN) is the most common microvascular complication in patients with T2DM (Vanaclocha-Espi et al., 2019). Its main clinical manifestations are mesangial matrix hyperplasia (Zhang et al., 2020), increased glomerular filtration rate (Hansen et al., 2002), and thickened basement membrane (Truong et al., 2019). It was reported that the disorder of glucose metabolism caused by T2DM can promote oxidative stress responses and induce the apoptosis of renal tubular epithelial cells, which are the main pathological mechanisms leading to DN (Calabrese et al., 2007; Rani et al., 2016). Studies have proven that bariatric surgery can effectively downregulate the disorder of glucose metabolism and lipid metabolism in the high-glucose environment caused by T2DM and inhibit the accumulation of oxidative stress products (Lone et al., 2020). However, whether bariatric surgery could improve the type II diabetes accompanying complications through regulating glycemia is still not clarified.

Bariatric surgery was initially designed to treat morbid obesity. It was reported to improve insulin resistance (Gumbs et al., 2005; Rao et al., 2012); however, the mechanism remains unclear. Roux-en-Y gastric bypass surgery can effectively relieve T2DM and related metabolic complications while treating morbid obesity (Bjornstad et al., 2020; Vangoitsenhoven et al., 2020). In a long-term follow-up study, it was found that patients with T2DM and impaired glucose tolerance maintained normal blood glucose levels after gastric bypass surgery. Moreover, 1 year after bariatric surgery, the ratio of urinary albumin creatinine was decreased from 16.5 to 6 mg/g (Kiortsis and Christou, 2012). Heneghan’s team conducted 5 years of long-term follow-up of 52 DN patients undergoing bariatric surgery. Their results showed that the remission rate of DN in 5 years after surgery reached 58.3% (Heneghan et al., 2013), suggesting that bariatric surgery may improve diabetic nephropathy.

Peroxidase proliferation-activated receptor alpha (PPARα) plays an essential role in regulating glucose and lipid metabolism (Balakumar et al., 2009). The activation of PPARα was reported to improve diabetic nephropathy in db/db mice (Park et al., 2006). The expression level of PPARα was affected by the nutritional status, blood glucose, and insulin influence (Medina-Gomez et al., 2005; Smati et al., 2020). Therefore, bariatric surgery may lead to the change of PPARα expression, further improving insulin resistance. This study aimed to establish a diabetic nephropathy model and perform bariatric surgery to observe its effects on glucose metabolism, renal function, and PPARα expression and to explore the regulatory mechanism of PPARα, so as to provide a theoretical basis for clinical surgical prevention and treatment of diabetic renal injury.

## Materials and Methods

### Experimental Animals

Forty SPF-grade Sprague–Dawley (SD) male rats (8 weeks old) weighing 230–250 g were provided by the Laboratory Animal Department of China Medical University [production license no. SCXK(Liao)-2013-0001, user license no. SYXK(Liao)-2013-0007]. The experiment was carried out in the barrier system of the Department of Laboratory Animals, China Medical University. Rats were randomly divided into the following groups: sham operation group (control group, *n* = 10), diabetic nephropathy group (DN group, *n* = 10), and DN + duodenal–jejunal bypass surgery group (DJB group, *n* = 20, 50% of the rats died during surgery). The experiment was approved by the Experimental Animal Welfare and Ethics Committee of China Medical University (IACUC no. 2019101). The protocol diagram is shown in Supplementary Figure 1.

### DN Rat Model Establishment

Rats were randomly divided into two groups after 1-week adaptive feeding. The high-sugar/high-fat (HSHF) combined diet (SF03-020, 40 kJ/kg, containing 23% fat, 20% protein, and 42% sucrose supplied by Specialty Feeds, Australia) was administered to one group of rats (*n* = 30) to induce insulin resistance. Meanwhile, normal chow (12% of calories as fat) was given to the other group (*n* = 10) of rats as a control for 8 weeks. Rats’ food intake and body weights were recorded weekly. Streptozotocin (STZ) solution (35 mg/kg) was injected intraperitoneally into the group of rats after 8 weeks of HSHF diet to establish the T2DM model. The blood glucose level was monitored after 3 days post-injection, and a successful T2DM model establishment was considered if the random blood glucose is higher than 16.7 mmol/L. T2DM rats were further fed with a high-glucose and high-fat diet for 6 weeks to induce renal function injury.

### Duodenal–Jejunal Bypass Surgery

After the DN rat model was successfully established, DN rats were injected with 1% pentobarbital sodium solution (45 mg/kg) intraperitoneally for anesthetization. All of the following surgical operations were carried out in a UV-disinfected operating table (the procedures are shown schematically in Supplementary Figure 2). Firstly, laparotomy of about 4–5 cm in the midline of the abdomen was performed; the pylorus and duodenum were then exposed through the incision. The proximal intestinal canal of the Treitz ligament was separated after positioning the Treitz ligament. The duodenum was transected from about 1 cm below the pylorus and residual chyme was squeezed out. Another jejunum transection from about 15 cm of the distal end of the Treitz ligament was carried out and the residual chyme was squeezed out, followed by the duodenum–jejunum anastomosis operation. Finally, the jejunum was transected again at about 10 cm distal to the previous anastomosis. The jejunum–jejunum anastomosis was performed to check the anastomosis for leaks, mesentery torsion, bleeding or strangulation. Afterward, the abdominal cavity was flushed with gentamicin saline, followed by sutures of the peritoneum, muscle, and skin, layer by layer.

The food intake and body weights of each group were continuously monitored after the operation, and an oral glucose tolerance test (OGTT) was performed every 2 weeks. Rats’ blood samples were collected for the examination of the content of blood creatinine; meanwhile, the urine samples were collected for measurement of the 24-h urinary protein excretion rate (urinary albumin excretion rate, UAER) and the urine creatinine content. The rats in each group were euthanized after continuous monitoring for 8 weeks. Part of the kidney tissues was fixed in paraformaldehyde, and the rest of the tissue sections were stored in liquid nitrogen.

The glomerular filtration rate (GFR, in milliliters per minute) was calculated as follows: [Urine inulin concentration (μg/ml) × UV (ml/min)]/[Plasma inulin conc (μg/ml)] (Hinojosa-Laborde et al., 2015).

### Hematoxylin–Eosin Staining

Paraformaldehyde-fixed kidney tissues were dehydrated and embedded. Then, the sections were deparaffinized with xylene, followed by rehydration for 5 min in ethanol gradients. Next, the sections were rinsed with distilled water and dyed in hematoxylin solution for 5–10 min, followed with washing processes and differentiation for 30 s in 1% acetic acid. Later, ammonia solution was used to stain the sections for 1 min. After 30 s counterstaining with eosin/phloxine solution, the sections were dehydrated with 95% ethanol twice for 5 min each. Finally, the sections were treated with xylene, sealed with neutral resins, and the morphological structure of the kidney tissues was observed under a light microscope.

### Masson Staining

Sliced kidney sections were deparaffinized and rehydrated with gradient ethanol (100, 95, and 70%), followed by a thorough rinse with distilled water. Wiegert’s iron hematoxylin staining solution was applied onto the sections for 10 min and the sections were rinsed in running water for 10 min. Biebrich scarlet–acid fuchsin solution was then applied onto the sections for 15 min staining. Later, the sections were differentiated in phosphomolybdic–phosphotungstic acid solution for 15 min. Afterward, the sections were transferred into an aniline solution directly and stained for 5 min. After a thorough rinse with distilled water, the sections were dehydrated immediately in absolute ethanol and treated in xylene twice. Finally, the sections were mounted with neutral resins. Fibrosis of the kidney tissues was observed under a light microscope.

### Periodic Acid–Schiff Staining

Kidney sections were placed on the coverslip in a staining dish after deparaffinization and were fixed for 10 min, followed with the washing processes. Then, periodic acid solution was used for differentiation for 10 min. Another washing process was performed. After washing three times with running water, the sections were dehydrated with gradient ethanol (50, 70, 80, 95, and 100% ethanol). Finally, the sections were treated with xylene and embedded with neutral resins. The results were visualized under a light microscope.

### Enzyme-Linked Immunosorbent Assay

The concentrations of tumor necrosis factor alpha (TNF-α; SCA133Ra, USCN, United States), interleukin 1β (IL-1β; SEA563Ra, USCN, United States), and IL-6 (SEA079Ra, USCN, United States) in rat renal tissue and the *in vitro* cell culture medium, superoxide dismutase (SOD; SES134Ra, USCN, United States), malondialdehyde (MDA; CEA597Ge, USCN, United States), and myeloperoxidase (MPO; SEA601Ra, USCN, United States) in rat serum were detected by the ELISA kits; the operating manuals were strictly followed in this study. Specifically, the standards and samples were added into the enzymatic reaction plates. Then, the plates were incubated at 37∘C for 1 h, followed with washing processes with PBS-Tween 20 three times. The plates were then incubated for another 30 min after the addition of detection reagents A and B. Afterward, another round of washing processes was carried out five times. Of the 3,3,5,5′-tetramethylbenzidine (TMB) substrate solution, 90 μl was then added to each well and the stop solution was added after a 15-min reaction. The plates were examined under a microplate reader and the optical density (OD) values were detected and recorded.

### TUNEL Staining

A terminal deoxynucleotidyl transferase dUTP nick-end labeling (TUNEL) staining kit (C10619, Invitrogen, United States) was used to detect renal cell apoptosis. Renal tissues were deparaffinized with xylene twice (5 min each) after slicing. Then, the sections were dehydrated with gradient ethanol (100, 95, 80, 75, and 50%) and rinsed with distilled water. Later, the sections were incubated in proteinase K/10 mM Tris solution for 15–30 min at 37∘C. TUNEL detection solution was applied to each section and the sections were incubated for 60 min at 37∘C in the dark. After rinsing with phosphate-buffered saline (PBS) three times, the sections were sealed with the anti-fluorescence quenching mounting reagent. Finally, the sections were examined under a fluorescence microscope.

### Immunofluorescence

The kidney tissue sections were placed into the citrate buffer solution for antigen retrieval after being deparaffinized. After heating and boiling the solution for 5 min, the sections were incubated with 3% H_2_O_2_ at room temperature for 10 min. Primary antibodies against PPARα (MA1-822, Invitrogen, United States) and NF-κB (PA5-16545, Invitrogen, United States) were added to the sections for immunostaining, followed by the application of fluorescent-labeled secondary antibody. The sections were then incubated at room temperature for 1 h. The cell nucleus was stained with DAPI for 15 min. Finally, the kidney tissues of each group were visualized under a fluorescence microscope.

### ROS Fluorescence Staining

2′-7′-Dichlorofluorescin diacetate (DCFH-DA; #287810, Sigma, United States) was used for cytosolic reactive oxygen species (ROS) and DHR123 (#309825, Sigma, United States) for mitochondrial ROS detection. Propidium iodide (PI) was used as a counterstain to assess membrane integrity. DCFH-DA and DHR123 staining was carried out accordingly (Kiani-Esfahani et al., 2012). Briefly, the tissue sections were incubated with 0.5 μM DCFH-DA or 0.05 μM DHR123 at room temperature in a dark setting for 40 min and then observed under a fluorescent microscope.

### Western Blotting

The cryopreserved kidney tissues were homogenized with the addition of RIPA lysis buffer (89900, Thermo Scientific, United States) and protease inhibitor (36978, Thermo Scientific, United States). Protein samples were collected after centrifugation and were stored at −80∘C. The concentrations of the protein samples were detected with a bicinchoninic acid (BCA) protein assay kit (23235, Thermo Scientific, United States). Then, the protein samples were loaded into 10% SDS-PAGE gels for electrophoresis, followed by membrane transfer and the blocking process. The membranes were incubated at 4°C overnight with primary antibodies against AMPKα (ab131512, Abcam, United States), p-AMPKα (ab133448, Abcam, United States), PPARα (ab24509, Abcam, United States), NF-κB (ab16502, Abcam, United States), and β-actin (ab8227, Abcam, United States) as the loading control. Afterward, the horseradish peroxidase (HRP)-conjugated goat anti-rabbit secondary antibody (ab205718, Abcam, United States) was applied and the membranes were incubated at room temperature for 1 h. A SuperSignal^TM^ West Pico PLUS Chemiluminescent Substrate (34580, Thermo Scientific, United States) was used to detect the expression levels of the target proteins in an iBright^TM^ FL1500 Imaging System (A44115, Invitrogen, United States). The gray values were calculated by ImageJ software.

### Statistical Analysis

Statistical analysis was performed by using SPSS 18.0 (IBM SPSS Statistics). All data were represented as the mean ± standard deviation (SD). For data with normal distribution, two-way analysis of variance (ANOVA) was used for comparisons among multiple groups and the LSD *t*-test used for comparisons between two groups. For data with abnormal distribution, parametric tests (e.g., Friedman) was used for comparison.

## Results

### Bariatric Surgery Improves Glucose Metabolism in Diabetic Rats

**FIGURE 1:**
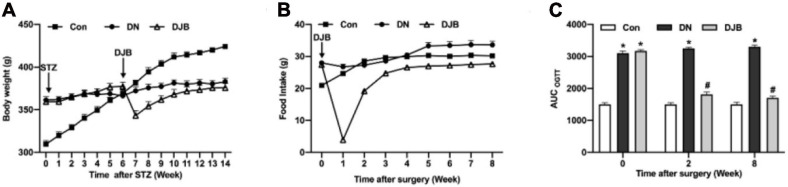
Bariatric surgery improved glucose metabolism in diabetic rats. Detections of food intake **(A)** and body weights **(B)** in each group of rats. **(C)** Oral glucose tolerance tests (OGTT) were conducted every 2 weeks for a total of 8 weeks of monitoring. All data represented the mean ± SD. *p < 0.05 [compared with the control (Con) group]; ^#^*p* < 0.05 [compared with the diabetic nephropathy (DN) group].

To study the effect of bariatric surgery on rats with diabetes, we established a useful type 2 diabetic rat model using high-fat and high-sugar (HFHS) diet following a low-dose STZ treatment (Srinivasan et al., 2005). The food intake (Figure 1A) and body weights (Figure 1B) of the rats were both significantly increased with the administration of a high-fat/high-sugar diet. STZ was intraperitoneally injected into rats on the HFHS diet after an 8-week feeding; the rats were then continually fed with the HFHS diet for 6 weeks to establish the DN model, and the body weight stopped growing by then. After bariatric surgery, 10 DN rats died. The food intake decreased significantly in the DJB group of rats and returned to the preoperative level at 3 weeks post-surgery. After 4 weeks, there was no significant difference in the food intake between the groups. The body weights of rats in the DJB group decreased to the lowest level 1 week post-surgery and recovered to the preoperative level on the second week. The body weights were monitored for 8 weeks post-operation and there were no significant differences among the three groups on the eighth week. The OGTT test showed that the glucose tolerance of rats in the DN and DJB groups was abnormal after the DJB surgery (Figure 1C, week 0), and the area under the curve (AUC) was higher than that of the control group. The glucose tolerance of the DJB rats was improved after 2 weeks of bariatric surgery (Figure 1C, weeks 2 and 8), suggesting that bariatric surgery can improve glucose metabolism in diabetic rats.

### Bariatric Surgery Improves Renal Dysfunction in Diabetic Rats

**FIGURE 2:**
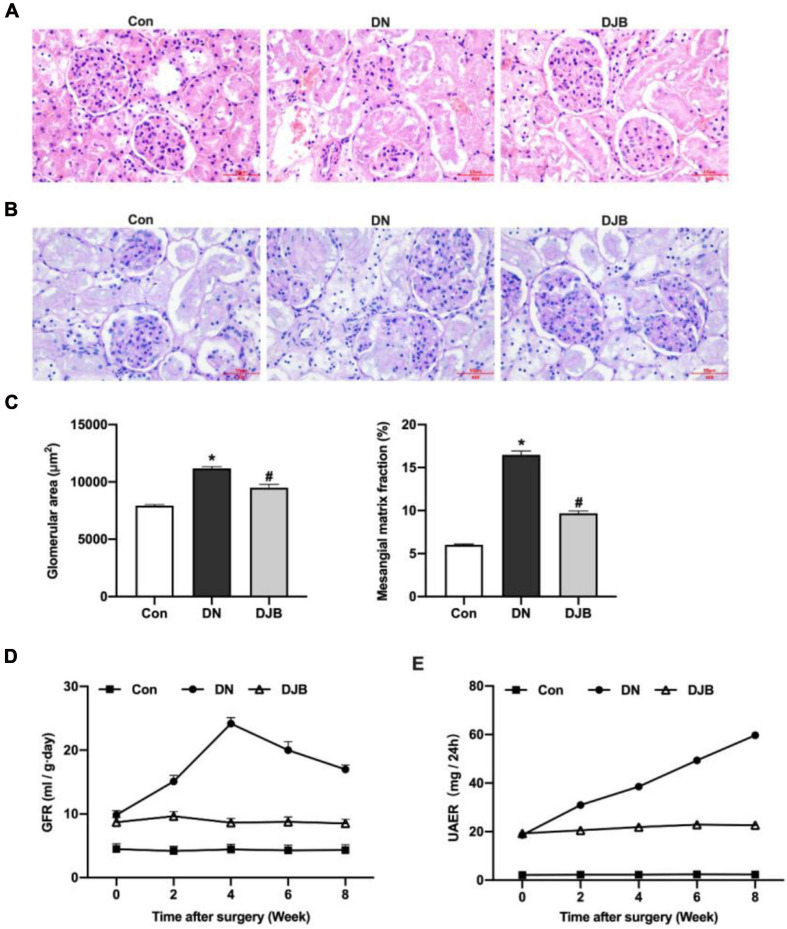
Bariatric surgery improved renal dysfunction in diabetic rats. Representative photomicrographs of the kidney sections observed with hematoxylin–eosin (H&E) staining **(A)** and periodic acid–Schiff (PAS) staining **(B)** (*scale bar*, 50 μm). Glomeruli in the kidney sections were visualized with H&E staining. The area of the mesangial matrix (*pink*/*purple* between the capillary loops) was seen on the PAS stain for collagen. **(C)** Quantification and comparison of the glomerular area and extra-mesenchymal matrix fraction. The histograms represent data from examining 25–50 individual glomeruli in sections from four individual mice in each group. Glomerular filtration rate (GFR) **(D)** and urinary albumin excretion rate (UAER) **(E)** monitored in 8 weeks. All data represented the mean ± SD. *p < 0.05 [compared with the control (Con) group]; ^#^*p* < 0.05 [compared with the diabetic nephropathy (DN) group].

To test the effect of bariatric surgery on renal function in diabetic rats, the rat tissues of the different groups were stained and compared. A widened mesangial area and an increased mesangial matrix were observed by hematoxylin–eosin (H&E) staining and periodic acid–Schiff (PAS) staining in the DN compared to the Con group (Figures 2A–C). The GFR was highest at week 4, then decreased significantly in the DN group (Figure 2D). UAER increased continually in 8 weeks (Figure 2E). After bariatric surgery in DM rats, the above pathological changes were relieved and the relevant diagnostic indicators were ameliorated.

### Bariatric Surgery Inhibits Oxidative Stress Injury Through Activating PPARα

**FIGURE 3:**
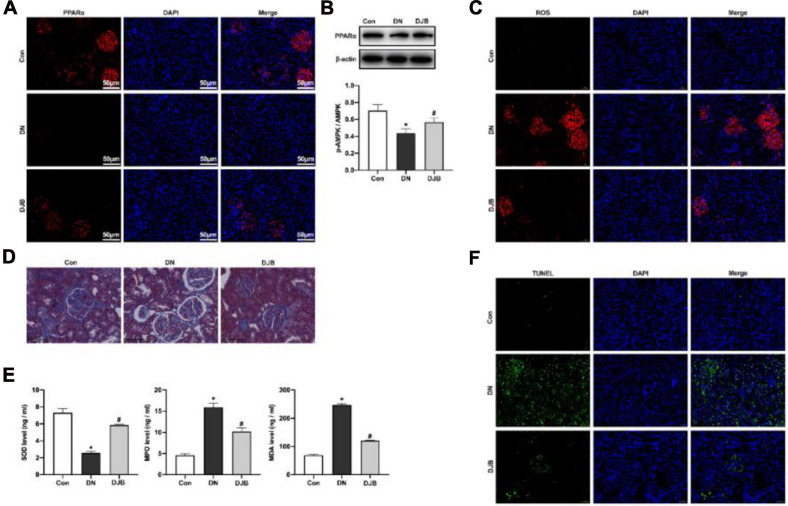
Bariatric surgery inhibited oxidative stress injury through activating PPARα. The expression of PPARα was detected by immunofluorescence (IF; *scale bar*, 50 μm) **(A)** and Western blotting **(B)**. **(C)** Mitochondrial ROS detected by DHR123 staining (*scale bar*, 50 μm). **(D)** Fibrosis of the kidney tissues observed with Masson staining (*scale bar*, 100 μm). **(E)** Quantification of the oxidation-related factors (SOD, MDA, and MPO) in rat serum by ELISA. **(F)** Renal apoptosis detected by TUNEL staining (*scale bar*, 50 μm). All data represented the mean ± SD. *p < 0.05 [compared with the control (Con) group]; ^#^*p* < 0.05 [compared with the diabetic nephropathy (DN) group]. ROS, reactive oxygen species; SOD, superoxide dismutase; MDA, malondialdehyde; MPO, myeloperoxidase.

PPARα is a nuclear hormone receptor involved in regulating lipid metabolism, oxidative stress, and glucose homeostasis. The expression of PPARα was downregulated in rats of the DN model and was elevated after bariatric surgery (Figures 3A,B), indicating that bariatric surgery could activate PPARα expression in DM rats. The content of mitochondrial-derived ROS in the kidney tissues in the DJB group was decreased compared to that in the DN group, as detected by DHR123 staining (Figure 3C). We also examined cytosolic ROS by DCFH-DA staining and observed similar results (Supplementary Figure 3). Moreover, the fibrosis of the kidney tissues was promoted in the DN group, as shown by Masson’s trichrome staining (Figure 3D). A decreased level of SOD in serum, along with higher levels of MDA and MPO, was also detected in rats in the DN group (Figure 3E). In contrast, bariatric surgery can effectively suppress the oxidative stress responses. Furthermore, renal apoptosis was detected by TUNEL staining, with the results showing that bariatric surgery could inhibit the apoptosis of renal cells (Figure 3F).

### Bariatric Surgery Reduces NF-κB p65 Level to Inhibit Inflammation in Diabetic Rats

**FIGURE 4:**
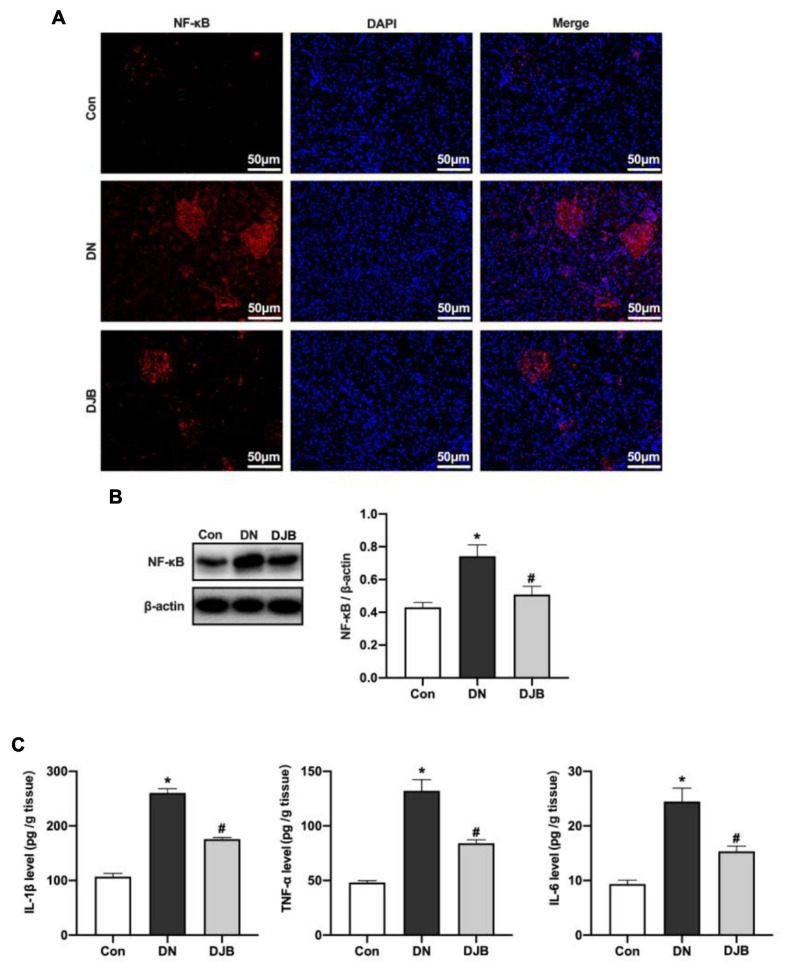
Bariatric surgery reduced the expression of NF-κB p65 to inhibit inflammation in diabetic rats. The expression of NF-κB was detected by immunofluorescence (IF; *scale bar*, 50 μm) **(A)** and Western blotting **(B)**. **(C)** Levels of the inflammatory factors (TNF-α, IL-1β and IL-6) in rat renal tissues examined by ELISA. All data represented the mean ± SD. *p < 0.05 [compared with the control (Con) group]; ^#^*p* < 0.05 [compared with the diabetic nephropathy (DN) group].

Previous studies have proven that activating PPARα can increase the responsive ability of liver fatty acids against oxidative stress and downregulate the NF-κB pathway, leading to anti-inflammatory responses. In the DN rat model, the expression of NF-κB p65 was increased (Figures 4A,B), and increased levels of inflammatory factors in the kidney tissues were detected (Figure 4C). With bariatric surgery, the expressions of the above protein and inflammatory factors were suppressed, leading to the inhibition of the inflammatory response.

### Bariatric Surgery Promoted the Phosphorylation of AMPKα *in vivo*: Phosphorylation of AMPKα Could Inhibit the Inflammatory Responses in High-Glucose-Cultured HK-2 Cells by Activating PPARα and Downregulating NF-κB *in vitro*

**FIGURE 5:**
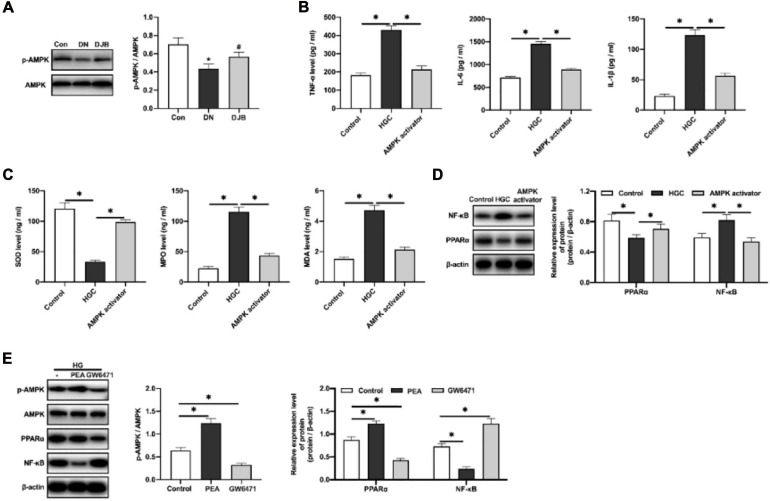
AMPKα inhibited the inflammatory responses in the high-glucose-cultured HK-2 cells by activating PPARα and downregulating NF-κB. **(A)** Adenosine 5′-monophosphate (AMP)-activated protein kinase (AMPK) and phosphorylated AMPK detected by Western blotting in rat renal tissues. Levels of the inflammatory factors **(B)** and oxidation-related factors **(C)** in the cell culture supernatants were quantified by ELISA. **(D)** Expression levels of PPARα and NF-κB in HK-2 cells detected by Western blotting. **(E)** Protein expression detected by Western blotting. All data represented the mean ± SD. ^∗^*p* < 0.05 [compared with the control (Con) group]; ^#^*p* < 0.05 [compared with the diabetic nephropathy (DN) group].

It is reported that the adenosine 5′-monophosphate (AMP)-activated protein kinase (AMPK) signaling pathway plays an essential role in lipid metabolism in the obese liver (Li et al., 2018), and it has been confirmed that AMPK agonists can elevate the expression of PPARα in obese cells to improve the oxidative stress progress of lipid metabolism (Kim et al., 2017). Similarly, we found that bariatric surgery could promote the phosphorylation of AMPKα in the kidney tissues of DM rats (Figure 5A). To explore the regulatory mechanisms of AMPKα in anti-inflammatory responses, an *in vitro* experiment of HK-2 cells cultured in a high-glucose environment was carried out. As a result, increased levels of inflammatory factors (TNF-α, IL-1β, and IL-6) in cell supernatants were detected (Figure 5B). The level of the oxidative stress factor, SOD, was decreased, along with the increased contents of MDA and MPO (Figure 5C). With the intervention of AMPKα agonists, the expression of PPARα was increased and the expression of NF-κB was decreased (Figure 5D), indicating that the inflammatory responses and oxidative stress injury were suppressed.

To further explore the regulatory relations among PPARα, AMPKα, and NF-κB, the PPARα agonist (PEA, 0.2 μmol/kg) and the PPARα antagonist (GW6471, 0.2 μmol/kg) were applied to the high-glucose-cultured HK-2 cells. The results showed that PPARα can promote the phosphorylation of AMPKα and inhibit the expression of NF-κB. Converse results were observed with the administration of the PPARα antagonist (Figure 5E). The above results suggested that bariatric surgery can activate the AMPKα pathway, promote the expression of PPARα, improve the oxidative stress responses, and downregulate the downstream NF-κB pathway, leading to the reduction of inflammatory responses.

## Discussion

Diabetic kidney injury is the most common diabetic complication and the most common cause of end-stage renal disease (ESRD) (Burrows et al., 2017). In this study, with a HFHS diet and a low-dose STZ (35 mg/kg) intraperitoneal injection, a T2DM rat model was constructed. The HFD/STZ rat model of type 2 diabetes was first reported in 2,000 by Reed et al. (2000). They fed 7-week-old Sprague–Dawley rats a diet with 40% kcal fat for 2 weeks. Subsequently, the animals were dosed once with STZ (50 mg/kg, i.v.). Later, another HFD/STZ rat model was generated by using a low dose of STZ (35 mg/kg, i.p.) by Srinivasan et al. (2005). The HFD/STZ model was then further modified by Zhang et al., wherein the STZ treatment comprised multiple low doses of STZ (2 × 30 mg/kg, i.p.) instead of a single dose (Zhang et al., 2008). After these three key publications, many versions of HFD/STZ rats appeared in the literature (see review article by Skovsø (Skovso, 2014)). Different diets were used to feed rats, and the duration of the diet before STZ treatment ranges from 1.5 to 12 weeks. For example, Hu et al. fed rats with a high-fat diet (26% sugar, 15.2% protein, and 58% fat) for 12 weeks before STZ treatment (35 mg/kg, i.p.) to establish a type 2 diabetic rat model (Hu et al., 2013). Yang et al. fed rats with a high-fat/high-sugar diet (40% sugar, 13% protein, and 40% fat) similar to that in our study for 8 weeks before STZ (15 mg/kg, i.v.) to generate a type 2 diabetic rat model (Yang et al., 2003). Parveen et al. fed rats with a high-fat/high-sugar diet (41% sugar, 18% protein, and 40% fat) for 2 weeks before STZ (40 mg/kg, i.p.) to establish a type 2 diabetic rat model (Parveen et al., 2010). The HFD/STZ type 2 diabetic rat model closely mimics the natural history and progression of the disease events, as well as the metabolic characteristics of human type 2 diabetes (Reed et al., 2000; Srinivasan et al., 2005). The aim of this study was to explore the protective effects and the regulatory mechanisms of bariatric surgery on kidney injury in diabetic rats (Supplementary Figure 4).

Bariatric surgery, also known as “metabolic surgery,” is the primary intervention to reducing blood glucose in patients with type 2 diabetes and obesity, which is considered as a standard treatment of diabetes (Docherty and Le Roux, 2020). Studies have shown that bariatric surgery can have a powerful, beneficial effect on all aspects of glucose homeostasis, which is independent of weight loss. In addition, clinical trials have shown that bariatric surgery is more effective than any lifestyle and/or pharmaceutical intervention in improving the blood glucose levels and reducing the incidence of microvascular complications and death (Medina-Gomez et al., 2005). It was reported that the all-cause mortality among patients who underwent bariatric surgery was reduced, including most of the reduction in the mortality rate of diabetes-related deaths (Parrott et al., 2017). Therefore, bariatric surgery may be an effective measure for the prevention and treatment of DN. In this study, bariatric surgery was performed on DN rats, and the glucose tolerance of the rats was significantly improved after 2 weeks. Metabolic damage caused by hyperglycemia is the key cause of DN development, including glomerular basement membrane thickening and mesangial membrane dilatation (Al-Hilali et al., 2020). Bariatric surgery reduced the renal histopathological damage and improved the glomerular filtration function. After bariatric surgery, the GFR was decreased slowly and the promotion of UAER was inhibited, indicating that bariatric surgery could improve the renal function of DN rats.

Hyperglycemia-induced vascular complications are associated with extensive tissue and organ damage (Aronson and Rayfield, 2002). A hyperglycemic microenvironment as the initial factor can cause kidney cell metabolism disorder and produce excess amounts of reactive oxygen species (Kolluru et al., 2012). The oxidative stress damaged cells accumulated in the kidney, leading to kidney tissue fibrosis and renal tissue apoptosis (Calabrese et al., 2007). PPARα, as an important ligand-activated transcription factor, plays an important role in the regulation of oxidative stress in various disease models (Kume et al., 2008). Studies have shown that the PPARα agonist Fenofibrate can improve renal function by inhibiting the oxidative stress, inflammatory response, and apoptosis in diabetic nephropathy rats (Hou et al., 2010; Yaribeygi et al., 2018). In this study, we found that bariatric surgery can activate PPARα, inhibit ROS generation, inhibit oxidative stress injury, and thereby decrease the renal apoptosis rate. Therefore, the protective effect of bariatric surgery on DN kidney is related to the activation of PPARα to inhibit oxidative stress.

The oxidative damage of DNA will promote the release of inflammatory factors. Long-term chronic inflammatory environment in kidney tissues could recruit the pro-inflammatory factors, leading to an inflammatory cascade reaction (Sato and Yanagita, 2017; Khan et al., 2020). A study has shown that the activation of PPARα can improve the oxidative stress process of lipid metabolism, enhance the oxidative stress response capacity of fatty acids in the liver, downregulate the downstream NF-κB pathway, and reduce the inflammatory response (Kim et al., 2017). This is consistent with the results of bariatric surgery in this study, which could reduce the oxidative stress injury and inhibit the inflammatory response in DN. AMPK is the upstream factor of PPARα, which regulates a variety of transcription factors related to liver lipid metabolism (Ren et al., 2019). It has been demonstrated in obese cells that the AMPK activator can increase the expression of PPARα and improve the oxidative stress process of lipid metabolism (Li et al., 2018). We confirmed *in vitro* that the AMPK agonist can promote the upregulation of PPARα expression. In contrast, PPARα antagonism could inhibit the AMPK phosphorylation and downregulate NF-κB expression.

In conclusion, bariatric surgery could activate the AMPK pathway, promote the expression of PPARα, improve oxidative stress, downregulate the downstream NF-κB pathway, and reduce the inflammatory response. However, there are limitations in our study. We did not demonstrate any effect of bariatric surgery on diabetic nephropathy independent of the known beneficial effect of improved glycemia. Moreover, a detailed study in the future is required to thoroughly characterize the pathophysiology of this new model.

## Data Availability Statement

The original contributions presented in the study are included in the article/Supplementary Material, further inquiries can be directed to the corresponding author/s.

## Ethics Statement

The animal study was reviewed and approved by China Medical University.

## Author Contributions

H-WJ and X-TB designed the study. P-YZ and T-YZ performed the experiments. J-YH collected and analyzed data. YZ wrote the manuscript. All authors contributed to the article and approved the submitted version.

## Conflict of Interest

The authors declare that the research was conducted in the absence of any commercial or financial relationships that could be construed as a potential conflict of interest.

## References

[B1] Al-Hilali K. A. Mosa M. J. Hussein A. A. (2020). The role of hyperglycemia and coexisting hypertension in the development of diabetic nephropathy in Type II diabetes mellitus. *Medico Legal Update* 20 1161–1167.

[B2] Aronson D. Rayfield E. J. (2002). How hyperglycemia promotes atherosclerosis: molecular mechanisms. *Cardiovasc. Diabetol.* 1:1. 10.1186/1475-2840-1-1 12119059 PMC116615

[B3] Balakumar P. Arora M. K. Singh M. (2009). Emerging role of PPAR ligands in the management of diabetic nephropathy. *Pharmaco. Res.* 60 170–173. 10.1016/j.phrs.2009.01.010 19646656

[B4] Bjornstad P. Nehus E. van Raalte D. (2020). Bariatric surgery and kidney disease outcomes in severely obese youth. *Semin. Pediatr. Surg.* 29:150883. 10.1016/j.sempedsurg.2020.150883 32238288 PMC7125208

[B5] Burrows N. R. Hora I. Geiss L. S. Gregg E. W. Albright A. (2017). Incidence of end-stage renal disease attributed to diabetes among persons with diagnosed diabetes—United States and Puerto Rico, 2000–2014. *MMWR Morb. Mortal. Weekly Rep.* 66:1165. 10.15585/mmwr.mm6643a2 29095800 PMC5689212

[B6] Calabrese V. Mancuso C. Sapienza M. Puleo E. Calafato S. Cornelius C. (2007). Oxidative stress and cellular stress response in diabetic nephropathy. *Cell Stress Chaperon.* 12:299. 10.1379/CSC-270.1 18229449 PMC2134792

[B7] Docherty N. G. Le Roux C. W. (2020). Bariatric surgery for the treatment of chronic kidney disease in obesity and type 2 diabetes mellitus. *Nat. Rev. Nephrol.* 16 709–720. 10.1038/s41581-020-0323-4 32778788

[B8] Guay C. Regazzi R. (2013). Circulating microRNAs as novel biomarkers for diabetes mellitus. *Na. Rev. Endocrinol.* 9:513. 10.1038/nrendo.2013.86 23629540

[B9] Gumbs A. A. Modlin I. M. Ballantyne G. H. (2005). Changes in insulin resistance following bariatric surgery: role of caloric restriction and weight loss. *Obes. Surg.* 15 462–473. 10.1381/0960892053723367 15946423

[B10] Hansen H. P. Hovind P. Jensen B. R. Parving H. H. (2002). Diurnal variations of glomerular filtration rate and albuminuria in diabetic nephropathy. *Kidney Intern.* 61 163–168. 10.1046/j.1523-1755.2002.00092.x 11786097

[B11] Heneghan H. M. Cetin D. Navaneethan S. D. Orzech N. Brethauer S. A. Schauer P. R. (2013). Effects of bariatric surgery on diabetic nephropathy after 5 years of follow-up. *Surg. Obes. Relat. Dis.* 9 7–14. 10.1016/j.soard.2012.08.016 23211651

[B12] Hinojosa-Laborde C. Jespersen B. Shade R. (2015). Physiology lab demonstration: glomerular filtration rate in a rat. *J. Vis. Exper.* 2015:e52425. 10.3791/52425 26274567 PMC4545064

[B13] Hou X. Shen Y. H. Li C. Wang F. Zhang C. Bu P. (2010). PPARα agonist fenofibrate protects the kidney from hypertensive injury in spontaneously hypertensive rats via inhibition of oxidative stress and MAPK activity. *Biochem. Biophys. Res. Commun.* 394 653–659. 10.1016/j.bbrc.2010.03.043 20226762

[B14] Hu S. H. Jiang T. Yang S. S. Yang Y. (2013). Pioglitazone ameliorates intracerebral insulin resistance and tau-protein hyperphosphorylation in rats with type 2 diabetes. *Exp. Clin. Endocrinol. Diabetes* 121 220–224. 10.1055/s-0032-1333277 23512416

[B15] Khan T. H. Ganaie M. A. Alharthy K. M. Madkhali H. Jan B. L. Sheikh I. A. (2020). Naringenin prevents doxorubicin-induced toxicity in kidney tissues by regulating the oxidative and inflammatory insult in Wistar rats. *Archiv. Physiol. Biochem.* 126 300–307. 10.1080/13813455.2018.1529799 30406686

[B16] Kiani-Esfahani A. Tavalaee M. Deemeh M. R. Hamiditabar M. Nasr-Esfahani M. H. (2012). DHR123: an alternative probe for assessment of ROS in human spermatozoa. *Syst. Biol. Reprod. Med.* 58 168–174. 10.3109/19396368.2012.681420 22545706

[B17] Kim S. M. Lee B. L. An H. J. Kim D. H. Park K. C. Noh S.-G. (2017). Novel PPARα agonist MHY553 alleviates hepatic steatosis by increasing fatty acid oxidation and decreasing inflammation during aging. *Oncotarget* 8: 46273. 10.18632/oncotarget.17695 28545035 PMC5542266

[B18] Kiortsis D. N. Christou M. A. (2012). Management of obesity-induced kidney disease: a critical review of the literature. *Obes. Facts* 5 821–832. 10.1159/000345919 23207569

[B19] Kolluru G. K. Bir S. C. Kevil C. G. (2012). Endothelial dysfunction and diabetes: effects on angiogenesis, vascular remodeling, and wound healing. *Intern. J. Vasc. Med.* 2012:918267. 10.1155/2012/918267 22611498 PMC3348526

[B20] Kume S. Uzu T. Isshiki K. Koya D. (2008). Peroxisome proliferator-activated receptors in diabetic nephropathy. *PPAR Res.* 2008:879523. 10.1155/2008/879523 19277201 PMC2652581

[B21] Li L. He M. Xiao H. Liu X. Wang K. Zhang Y. (2018). Acetic acid influences BRL-3A cell lipid metabolism via the AMPK signalling pathway. *Cell. Physiol. Biochem.* 45 2021–2030. 10.1159/000487980 29529605

[B22] Lone A. Behl T. Kumar A. Makkar R. Nijhawan P. Redhu S. (2020). Renoprotective potential of dimethyl fumarate in streptozotocin induced diabetic nephropathy in Wistar rats. *Obes. Med.* 18:100237. 10.1016/j.obmed.2020.100237

[B23] Medina-Gomez G. Virtue S. Lelliott C. Boiani R. Campbell M. Christodoulides C. (2005). The link between nutritional status and insulin sensitivity is dependent on the adipocyte-specific peroxisome proliferator–activated receptor-γ2 isoform. *Diabetes* 54 1706–1716. 10.2337/diabetes.54.6.1706 15919792 PMC4304004

[B24] Park C. Zhang Y. Zhang X. Wu J. Chen L. Cha D. R. (2006). PPARα agonist fenofibrate improves diabetic nephropathy in db/db mice. *Kidney Intern.* 69 1511–1517. 10.1038/sj.ki.5000209 16672921

[B25] Parrott J. Frank L. Rabena R. Craggs-Dino L. Isom K. A. Greiman L. (2017). American society for metabolic and bariatric surgery integrated health nutritional guidelines for the surgical weight loss patient 2016 update: micronutrients. *Surg. Obes. Relat. Dis.* 13 727–741. 10.1016/j.soard.2016.12.018 28392254

[B26] Parveen K. Khan M. R. Mujeeb M. Siddiqui W. A. (2010). Protective effects of Pycnogenol on hyperglycemia-induced oxidative damage in the liver of type 2 diabetic rats. *Chem. Biol. Interact.* 186 219–227. 10.1016/j.cbi.2010.04.023 20433812

[B27] Rani V. Deep G. Singh R. K. Palle K. Yadav U. C. (2016). Oxidative stress and metabolic disorders: pathogenesis and therapeutic strategies. *Life Sci.* 148 183–193. 10.1016/j.lfs.2016.02.002 26851532

[B28] Rao R. Yanagisawa R. Kini S. (2012). Insulin resistance and bariatric surgery. *Obes. Rev.* 13 316–328. 10.1111/j.1467-789X.2011.00955.x 22106981

[B29] Reed M. J. Meszaros K. Entes L. J. Claypool M. D. Pinkett J. G. Gadbois T. M. (2000). A new rat model of type 2 diabetes: the fat-fed, streptozotocin-treated rat. *Metabolism* 49 1390–1394. 10.1053/meta.2000.17721 11092499

[B30] Ren L. Sun D. Zhou X. Yang Y. Huang X. Li Y. (2019). Chronic treatment with the modified Longdan Xiegan Tang attenuates olanzapine-induced fatty liver in rats by regulating hepatic de novo lipogenesis and fatty acid beta-oxidation-associated gene expression mediated by SREBP-1c, PPAR-alpha and AMPK-alpha. *J. Ethnopharmacol.* 232 176–187. 10.1016/j.jep.2018.12.034 30590197

[B31] Sato Y. Yanagita M. (2017). Resident fibroblasts in the kidney: a major driver of fibrosis and inflammation. *Inflamm. Regen.* 37:17. 10.1186/s41232-017-0048-3 29259716 PMC5725902

[B32] Skovso S. (2014). Modeling Type 2 diabetes in rats using high fat diet and streptozotocin. *J. Diabetes Investig.* 5 349–358. 10.1111/jdi.12235 25411593 PMC4210077

[B33] Smati S. Régnier M. Fougeray T. Polizzi A. Fougerat A. Lasserre F. (2020). Regulation of hepatokine gene expression in response to fasting and feeding: influence of PPAR-α and insulin-dependent signalling in hepatocytes. *Diabetes Metab.* 46 129–136. 10.1016/j.diabet.2019.05.005 31163275

[B34] Srinivasan K. Viswanad B. Asrat L. Kaul C. L. Ramarao P. (2005). Combination of high-fat diet-fed and low-dose streptozotocin-treated rat: a model for type 2 diabetes and pharmacological screening. *Pharmacol. Res.* 52 313–320. 10.1016/j.phrs.2005.05.004 15979893

[B35] Truong L. D. Gaber L. W. Khan F. (2019). Donor-related diabetic nephropathy: a comprehensive clinicopathological study. *Hum. Pathol.* 85 136–144. 10.1016/j.humpath.2018.10.032 30448223

[B36] Vanaclocha-Espi M. Ibáñez J. Molina-Barceló A. Valverde-Roig M. J. Pérez E. Nolasco A. (2019). Risk factors for severe complications of colonoscopy in screening programs. *Prevent. Med.* 118 304–308. 10.1016/j.ypmed.2018.11.010 30414944

[B37] Vangoitsenhoven R. Mulya A. Mosinski D. Brethauer S. A. Schauer P. R. Kirwan J. P. (2020). Effects of gastric bypass surgery on expression of glucose transporters and fibrotic biomarkers in kidney of diabetic fatty rats. *Surg. Obes. Relat. Dis.* 16 1242–1248. 10.1016/j.soard.2020.04.017 32505735 PMC8276306

[B38] Wilding J. (2014). The importance of weight management in type 2 diabetes mellitus. *Intern. J. Clin. Pract.* 68 682–691. 10.1111/ijcp.12384 24548654 PMC4238418

[B39] Yang J. Li G. Zhang F. Liu Y. Zhang D. Zhou W. (2003). Identification of variations of gene expression of visceral adipose and renal tissue in type 2 diabetic rats using cDNA representational difference analysis. *Chin. Med. J.* 116 529–533. 12875716

[B40] Yaribeygi H. Mohammadi M. T. Rezaee R. Sahebkar A. (2018). Fenofibrate improves renal function by amelioration of NOX-4, IL-18, and p53 expression in an experimental model of diabetic nephropathy. *J. Cell. Biochem.* 119 7458–7469. 10.1002/jcb.27055 29761900

[B41] Zhang M. Lv X. Y. Li J. Xu Z. G. Chen L. (2008). The characterization of high-fat diet and multiple low-dose streptozotocin induced type 2 diabetes rat model. *Exp. Diabetes Res.* 2008:704045. 10.1155/2008/704045 19132099 PMC2613511

[B42] Zhang M. Zhang Y. Xiao D. Zhang J. Wang X. Guan F. (2020). Highly bioavailable berberine formulation ameliorates diabetic nephropathy through the inhibition of glomerular mesangial matrix expansion and the activation of autophagy. *Eur. J. Pharmacol.* 873:172955. 10.1016/j.ejphar.2020.172955 32001218

