# Supplementary figures and images for: Protective Effects of Bariatric Surgery on Kidney Functions by Inhibiting Oxidative Stress Responses Through Activating PPARα in Rats With Diabetes
Source: Front Physiol. 2021 Jun 28;12:662666. doi: 10.3389/fphys.2021.662666 (PMC8275180; doi:10.3389/fphys.2021.662666)

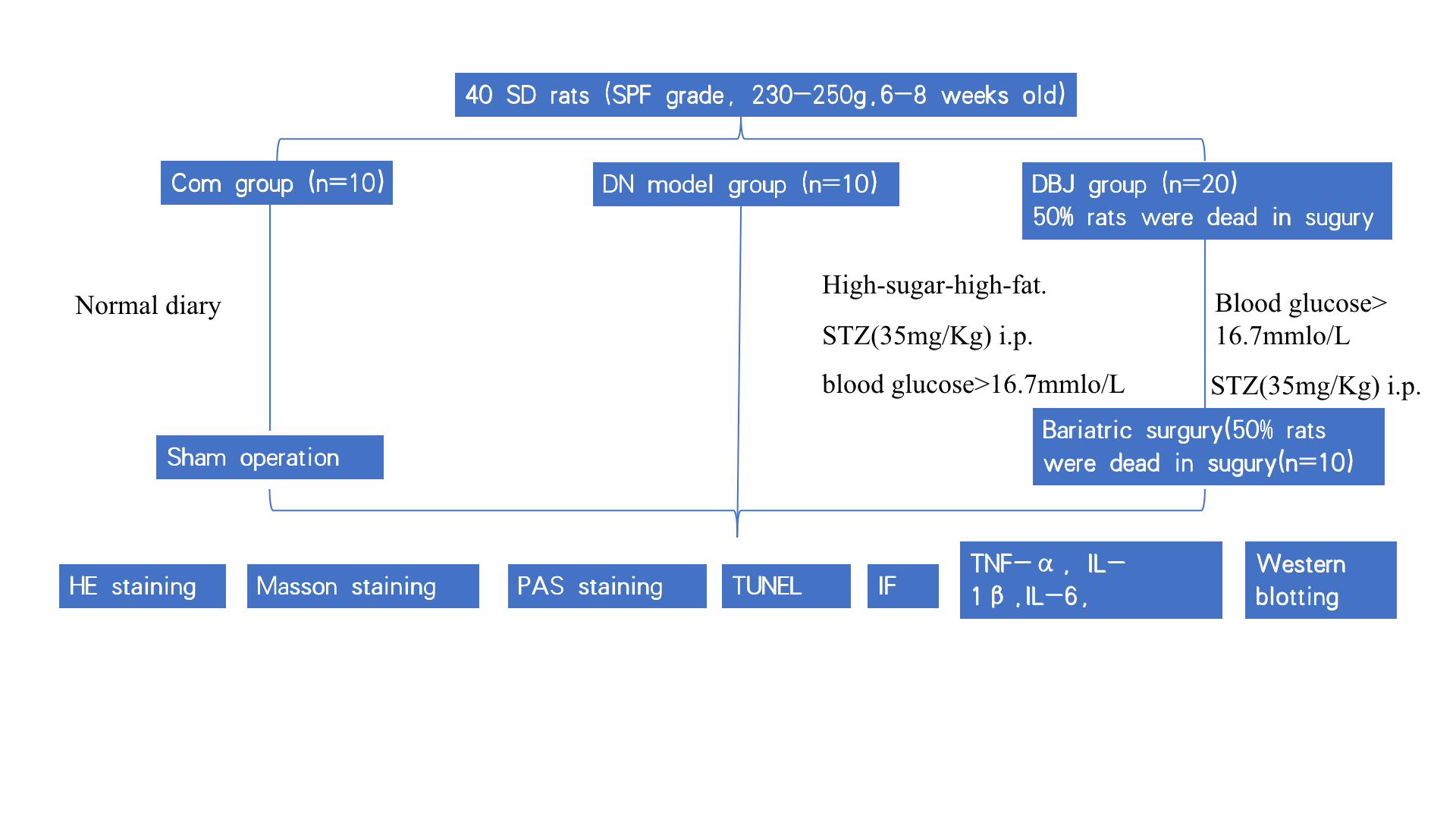

Supplement: Supplementary file 1 [file Image_1.tif]

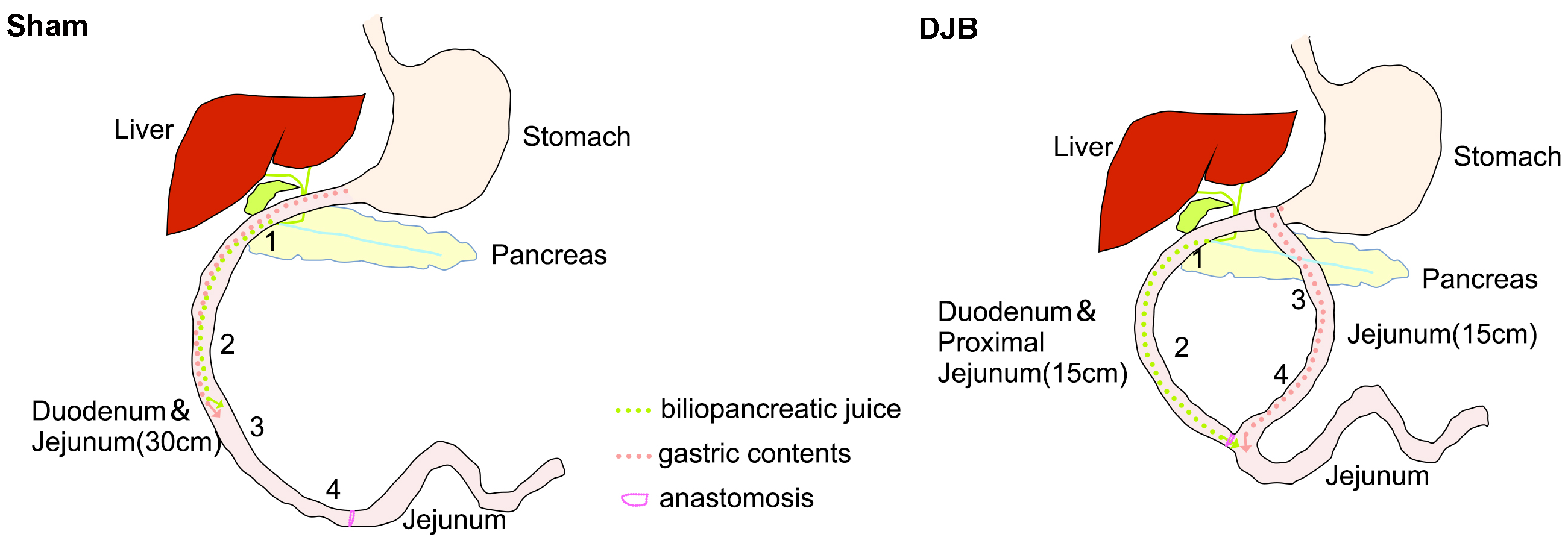

Supplement: Supplementary file 2 [file Image_2.tif]

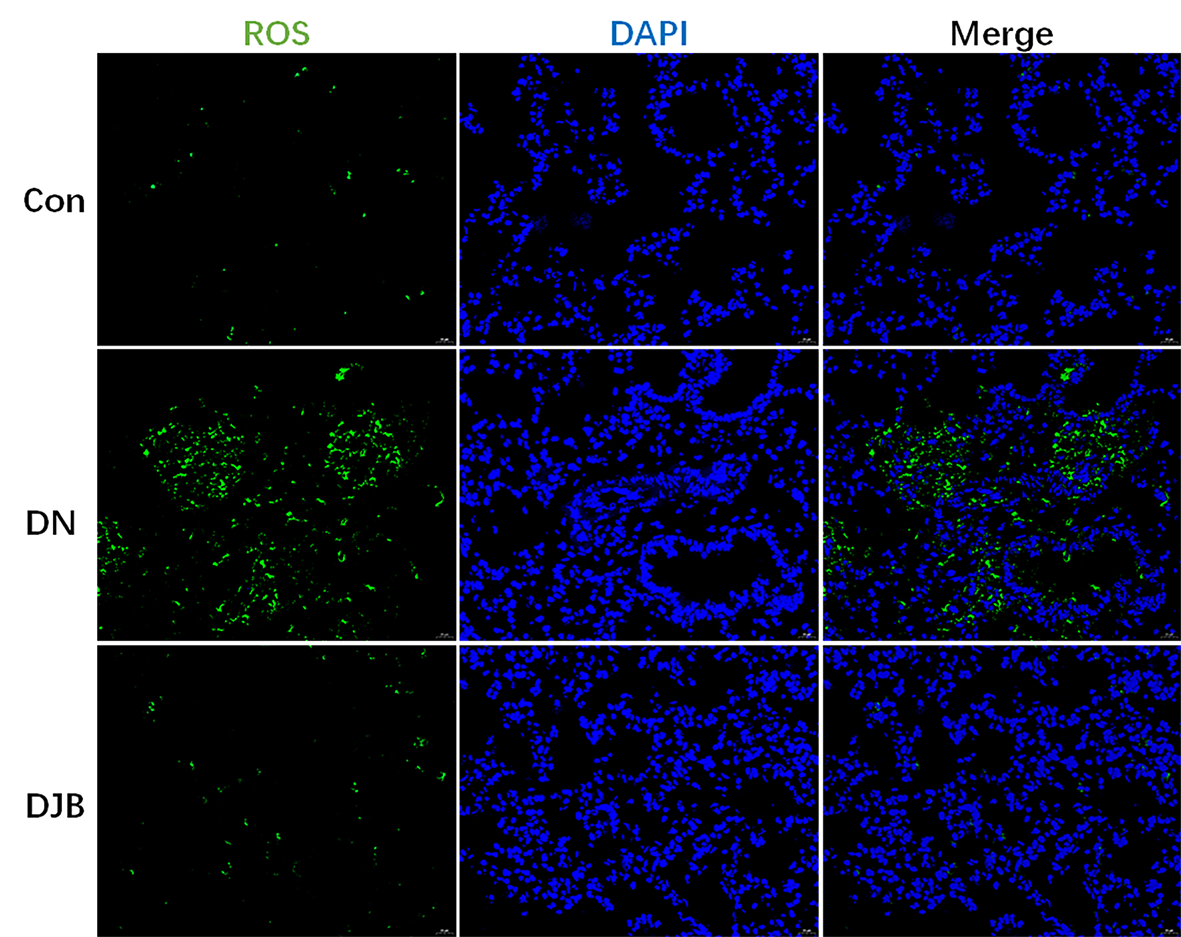

Supplement: Supplementary file 3 [file Image_3.tif]

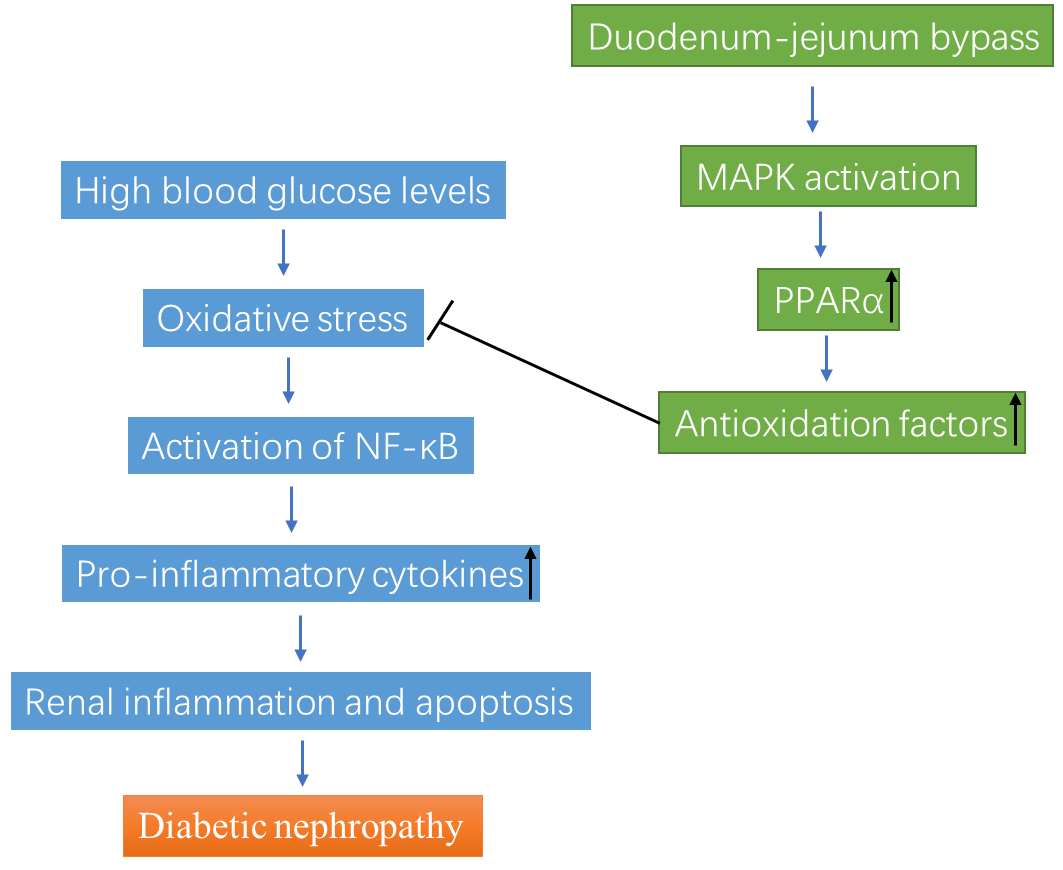

Supplement: Supplementary file 4 [file Image_4.png]
